# Clustering Patients With Chronic Cough Using Reported Sensations and Triggers: Results from the Triggers and Sensations Provoking Coughing Questionnaire

**DOI:** 10.1016/j.chest.2025.05.049

**Published:** 2025-07-18

**Authors:** Jenny King, Shannon Galgani, James Wingfield Digby, Joanne Mitchell, Kimberly Jane Holt, Rachel Jane Dockry, Sean M. Parker, Kathryn Prior, Chelsea Sawyer, Janelle Yorke, Jaclyn Ann Smith, Paul Anthony Marsden

**Affiliations:** a Division of Infection, Immunity and Respiratory Medicine, Faculty of Biological Sciences, The University of Manchester, Manchester, England; b Division of Psychology and Mental Health, Faculty of Biology, Medicine and Health, The University of Manchester, Manchester, England; c Cough Research Team, Manchester University NHS Foundation Trust, Manchester, England; d Department of Respiratory Medicine, Northumbria Healthcare NHS Foundation Trust, Northumbria, England; e Department of Respiratory Medicine, Lancashire Teaching Hospital NHS Foundation Trust, Preston, England; f School of Nursing, The Hong Kong Polytechnic University, Hong Kong; g Department of Respiratory Medicine, Manchester University NHS Foundation Trust, Manchester, England

**Keywords:** chronic cough, cluster analysis, phenotyping, quality of life, questionnaire development, refractory, sensations and triggers

## Abstract

**Background:**

Chronic cough (CC) is one of the most common symptoms reported to primary care and to respiratory outpatient clinics. The Triggers and Sensations Provoking Coughing (TOPIC) questionnaire is a 15-item questionnaire designed to capture sensations and triggers associated with CC in a fashion that discriminates between refractory CC (RCC) and other causes of CC.

**Research Questions:**

Does a relationship exist between TOPIC questionnaire scores and other measures of cough in patients referred to 3 specialist cough clinics? Can patients with CC be clustered based on reported sensations and triggers as captured by the TOPIC questionnaire?

**Study Design and Methods:**

This was a multicenter observational study. Data were collected including the Leicester Cough Questionnaire (LCQ) score, verbal cough severity, day and night cough visual analog scale score, 24-hour cough frequency, and TOPIC questionnaire results. Patterns of TOPIC questionnaire responses were explored using cluster analysis.

**Results:**

Baseline data were collected from 101 participants. Demographics of study participants were typical of patients with CC; 65% were female with a mean (SD) age of 59 (12.9) years. Baseline TOPIC questionnaire score correlated moderately negatively with baseline LCQ score (*r* = –0.59; *P* < .001), but not with other subjective or objective measures of cough. Hierarchical and K mean cluster analysis were used to group study participants into 4 distinct clusters based on triggers and sensations of cough as captured by the TOPIC questionnaire: (1) high sensations burden, (2) vocal triggers, (3) eating triggers, and (4) need to clear throat. These groups showed statistically significant differences in demographics and subjective measures of cough (*P* < .01).

**Interpretation:**

Patients with CC reported unpleasant somatic sensations and cough triggers captured by the TOPIC questionnaire, associated with lower LCQ scores and worse quality of life. Our results suggest that TOPIC questionnaire responses can be used to cluster patients into clinical phenotypes based on reported sensations and triggers.

FOR EDITORIAL COMMENT, SEE PAGE 1284

**Take-Home Points**

**Study Question:** Does a relationship exist between Triggers and Sensations Provoking Coughing (TOPIC) questionnaire results and other subjective and objective measures of cough in patients with chronic cough (CC) referred to UK specialist cough clinics?

**Results:** Baseline TOPIC questionnaire scores were associated with worse cough-related quality of life (QOL) as captured by the Leicester Cough Questionnaire (LCQ). Using the TOPIC questionnaire, study participants could be grouped into 4 distinct clusters based on triggers and sensations of cough: (1) high sensations burden, (2) vocal triggers, (3) eating triggers, and (4) need to throat clear.

**Interpretation:** Patients with CC reported unpleasant somatic sensations and cough triggers captured by the TOPIC questionnaire and associated with lower LCQ scores and worse QOL. Our results suggest that TOPIC questionnaire responses can be used to cluster patients into clinical phenotypes based on reported sensations and triggers.

Chronic cough (CC; cough lasting ≥ 8 weeks) is 1 of the most common symptoms reported to primary care and respiratory outpatient clinics.^1^, ^2^, ^3^, ^4^ CC can have many underlying causes, and the nature of the cough can differ depending on the cause. In some circumstances, it can become a pervasive disease despite addressing underlying treatable traits (eg, asthma), or if unexplained (no cause found), it can be referred to as refractory CC (RCC).^3^ Currently, RCC is a diagnosis of exclusion after systematic investigations and treatment trials for other causes of cough; however, this process is time consuming and costly, adding to the socioeconomic burden of this disease.^4^^,^^5^ A feature of RCC is that patients frequently describe similar, often unpleasant, somatic sensations (for example, an itch in the throat) and multiple cough triggers (eg, certain foods, perfumes, cold air, and talking or laughing) suggestive of hyperexcitability of the cough reflex.^5^, ^6^, ^7^ These commonly result in excessive coughing in response to ordinarily nontussive triggers (allotussia) and exaggerated cough responses to tussive triggers (hypertussia), referred to as *cough hypersensitivity*.^8^ Identifying these common features may have the potential to identify patients with RCC at an earlier stage, and thus those appropriate for therapy targeting neuronal mechanisms. Currently, no validated tool exists to capture and quantify these sensations and triggers adequately. Therefore, we developed the Triggers and Sensations Provoking Coughing (TOPIC) questionnaire, which evaluates the sensations and triggers provoking coughing that best discriminate RCC from cough associated with common respiratory diseases such as COPD, asthma, interstitial lung disease, and bronchiectasis. This newly developed 15-item TOPIC questionnaire was applied for the first time in the Sensations and Triggers of Coughing Pre- and Post-Treatment in Chronic Cough (StaRR) study. The StaRR study is an observational study designed to evaluate change in TOPIC questionnaire scores in new patients referred to specialist CC clinics after investigation and treatment of cough in accordance with international guidelines.^9^ It also aims to determine if these changes relate to changes in other subjective and objective measures of cough.

The aim of this analysis was to compare baseline TOPIC questionnaire scores with other subjective and objective measures of cough in consecutive new patients referred to 3 participating specialist UK cough clinics. We also explored whether the patterns of triggers and sensations of cough captured by the TOPIC questionnaire could be used to cluster patients.

## Study Design and Methods

This was a multicenter observational study that recruited 101 new patients with a history of CC from 3 United Kingdom-based specialist cough clinics. All eligible patients were offered the opportunity to participate after the first consultation. Patients were assessed in accordance with European Respiratory Society cough guidelines.^9^ Before the initiation of any new treatment, demographics, clinician diagnosis, cough duration, and verbal cough severity score were obtained and TOPIC questionnaire and Leicester Cough Questionnaire (LCQ) were completed. Participants were fitted with a 24-hour ambulatory cough monitor (VitaloJAK; Vitalograph, Ltd.) and completed day and night cough severity visual analog scales (VASs) at the end of this recording. The study was approved by the North West Greater Manchester South Research ethics committee (Identifier: 22/NW/0210).

### Patient Population

This study applied the TOPIC questionnaire to a broad population of patients older than 18 years with a CC (> 8 weeks), regardless of cause. Participants taking angiotensin converting enzyme (ACE) inhibitors, those who currently or formerly smoked heavily (> 20 pack-years or quit < 6 months ago), and those with a recent deterioration in pulmonary status (in last 4 weeks) were excluded from the study. Data were collected over a 19-month period from January 2023 through July 2024.

### Study Procedures

#### TOPIC Questionnaire

Participants were asked to complete the TOPIC questionnaire, a 15-item questionnaire in which participants graded specific cough sensations and triggers on a Likert scale (0-5; 0 = never to 5 = always). The total TOPIC questionnaire score ranges from 0 to 75, with higher scores indicating a higher frequency of sensations and specific triggers related to coughing. The questionnaire captures 2 domains, frequency of triggers resulting in coughing (scoring from 0-40) and sensations experienced because of coughing (scoring from 0-35).

#### Verbal Cough Severity Score

The participant gave a verbal cough score to describe their cough severity from 0 through 10 (0 is no cough and 10 is the worst cough imaginable).

#### Leicester Cough Questionnaire

Participants completed the LCQ, a 19-item questionnaire grading the impact of their cough on different aspects of their daily life on a scale of 1 through 7. The questionnaire gives a total score and 3 domain scores: physical, psychological, and social effects. A lower score is indicative of a worse cough-related quality of life (QOL).

#### Cough Monitoring

To measure objective cough rates, participants were fitted with a VitaloJAK ambulatory cough monitor. This was done at the first clinic appointment or remotely in certain circumstances within 7 days of consent and before starting treatment. If carried out remotely, instructions were provided on how to fit the cough monitor. The patient also was offered a telephone or video call with a researcher to guide them through the fitting. The device was worn for 24 hours, recording all sound via 2 microphones, 1 attached to the participant’s chest wall and 1 attached to the participant’s clothing. The device was set to stop recording automatically after 24 hours.

#### Visual Analog Score

The participant was provided with a 100-mm line on which they place a vertical line to rate the severity of their cough separately during the day and night (0 mm is no cough and 100 mm is the most severe cough ever experienced) to complete at the end of the 24-hour period when they were wearing the cough monitor.

### Statistical Analysis

Because this was an exploratory study, no formal power calculation was made. Study data were collected and managed using Research Electronic Data Capture tools.^10^^,^^11^ Data were analyzed using IBM SPSS Statistics version 25 software. Data distribution was assessed using the Kolmogorov-Smirnov test and by assessing skewness and kurtosis. Demographic data and subjective and objective measures of cough are presented as median (interquartile range) values for nonparametric data and as mean (SD) for parametric data unless otherwise stated. Cough count data were transformed logarithmically (base 10) before analysis and are presented as geometric means and compared using the unpaired *t* test for 2-way comparisons or analysis of variance testing for multiple group comparisons. Nonparametric data was compared using the Mann-Whitney *U* test and parametric data were compared using the unpaired Student *t* test. Nonparametric correlations were performed using Spearman rank correlation coefficient and parametric correlations were performed using Pearson correlations. Cronbach α test was used to assess internal consistency.

Exploratory factor analysis and principal component analysis were performed to identify patterns of TOPIC questionnaire responses. Hierarchical cluster analysis (Wards method) was used with the squared Euclidean distance to generate a dendrogram, to estimate the number of clusters in the data set, and to compare groups with those identified by the factor analysis. K means clustering then was used to label individual patients by cluster to compare demographics and subjective and objective measures of cough between clusters.

## Results

### Study Population and Characteristics

Baseline data were collected for 101 participants from the 3 specialist cough clinics participating in the study. The study was overrecruited to account for a participant who could not complete ambulatory cough monitoring as planned in the study design. The demographics of study participants were typical of patients with CC: 65% were female, with a mean (SD) age of 59 (12.9) years and age range of 18 to 84 years (Table 1). The patients reported a large range of cough duration (0.5-50 years). Participants predominantly had never smoked (63%), with normal baseline spirometry findings. Forty-seven percent of participants received a diagnosis of RCC at the first appointment. A further 27% needed further investigation, and for 26% of patients, it was believed that another disease process (for example asthma, COPD, or bronchiectasis) needed to be addressed.

**Table 1:** Demographic Characteristics

Demographic Characteristic	Baseline Data (N = 101)
Female sex	66 (65%)
Age, y	59 (12.9)
Cough duration, y, median (range)	6 (0.5-50)
Never smoked	64 (63%)
Duration of former smoking, pack-years	5 (0.7-13)
FEV_1_	
L	2.6 (2.1-3.1)
% predicted	101 (87-109)
FVC	
L	3.3 (2.8-4.0)
% predicted	103 (90-114)
FEV_1_ to FVC ratio	0.79 (0.71-0.84)
Diagnosis at first appointment	
RCC	47 (47%)
Other^a^	26 (26%)
Needs further investigation	27 (27%)

Data are reported as No. (%) or median (interquartile range) unless otherwise indicated. RCC = refractory (or unexplained) chronic cough.

a Any other condition for primary driver of cough, for example, bronchiectasis. Bronchiectasis, n = 7 (6.9%); interstitial lung disease, n = 2 (1.9%); and asthma requiring further optimization, n = 17 (16.8%).

### Measures of Cough

**Figure 1:**
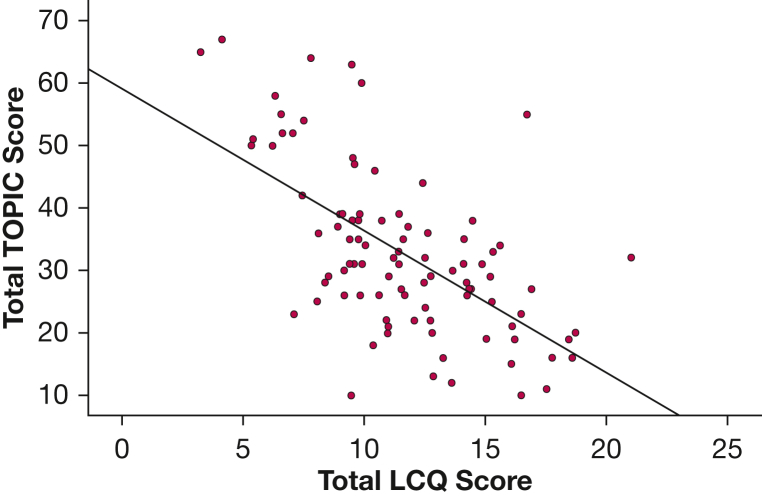
Scatterplot showing the negative correlation of TOPIC questionnaire score with LCQ score (*r* = –0.593; *P* < .001). LCQ = Leicester Cough Questionnaire; TOPIC = Triggers and Sensations Provoking Cough.

Subjective and objective measures of cough frequency and severity were highly variable among participants (Table 2). Baseline TOPIC questionnaire scores correlated moderately negatively with baseline LCQ score (*r* = –0.59; *P* < .001) (Fig 1), indicating that higher TOPIC questionnaire score was associated with worse QOL. Other subjective and objective measures of cough—including day VAS, night VAS, and verbal cough severity scores and 24-hour, awake, and asleep cough frequency—showed weak or minimal correlation with TOPIC questionnaire score (Table 3). A correlation matrix demonstrating the relationship among all subjective and objective measures of cough used within the study is shown in e-Table 1. Cronbach α demonstrated good internal consistency for TOPIC questionnaire items (0.857).

**Table 2:** Patient-Reported Outcomes and Objective Cough Frequency

Outcome Measure	Median (Interquartile Range)
Verbal cough severity score, /10	6 (5-8)
VAS score, mm	
Day	62 (39-72)
Night	35 (14-64)
LCQ	
Physical	4.3 (3.3-4.8)
Psychological	3.4 (2.7-4.6)
Social	3.5 (2.8-4.8)
Total	11.1 (9.2-14.2)
TOPIC questionnaire score	
Triggers	15 (10-21)
Sensations	16 (13-21)
Total	31 (25-41)
Cough frequency, coughs/h	
Awake	22.6 (10.3-36.1)
Asleep	1.6 (0.5-5.9)
24-hour	16.1 (7.0-27.0)

LCQ = Leicester Cough Questionnaire; TOPIC = Triggers and Sensations Provoking Cough; VAS = visual analog scale.

**Table 3:** Spearman Rank Correlation Coefficients of Total TOPIC Questionnaire Scores With Other Objective and Subjective Measures of Cough

Outcome Measure	Correlations With TOPIC Questionnaire Score	
	*r* Value	*P* Value
LCQ	–0.593	< .001
VAS score		
Day	**0.32**	**.005**
Night	0.19	.103
Verbal cough severity score	0.21	.051
Cough frequency		
24-hour	0.245	.051
Awake	**0.252**	**.016**
Asleep	0.099	.355

Boldface indicates statistically significant values. LCQ = Leicester Cough Questionnaire; TOPIC = Triggers and Sensations Provoking Cough; VAS = visual analog scale.

**Figure 2:**
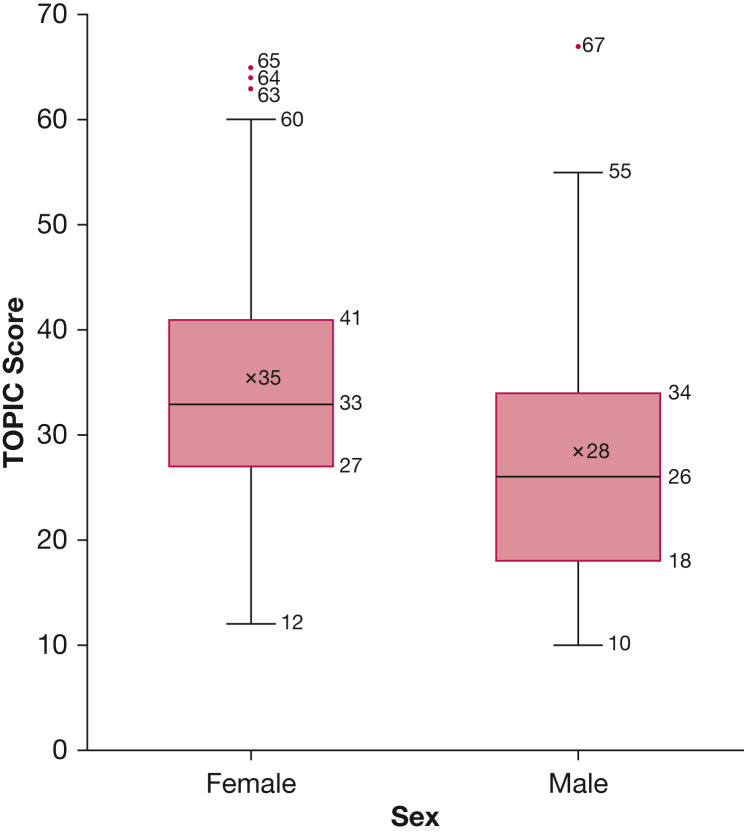
Box-and-whisker plot showing sex differences for TOPIC questionnaire scores. *P* = .016. TOPIC = Triggers and Sensations Provoking Coughing.

Female patients showed significantly higher TOPIC questionnaire scores (34.5 vs 26; *P* = .016) (Fig 2). They also showed lower LCQ scores (10.2 vs 12.6; *P* = .018) and higher cough frequency during sleep (median, 3 [interquartile range, 1-8] vs 1 [interquartile range, 0-4]; *P* = .046) compared with male patients (Table 2). Despite this, no significant difference was found in day VAS, night VAS, or verbal cough severity scores or awake and 24-hour cough frequency. Correlation coefficients for all subjective and objective measures of cough were not significantly different when the data were split and compared by sex. Age did not influence TOPIC questionnaire score significantly on univariate testing (*P* = .217).

### Cluster Analysis

Hierarchical cluster analysis produced a dendrogram that suggested that patients could be categorized into 4 main clusters based on patterns of sensations and triggers (e-Fig 1). K means cluster analysis determined the cluster membership of each participant. Demographic variables and measures of cough for each cluster were compared using nonparametric analyses (Table 4). This demonstrated 4 main clusters. Cluster 1 reported predominantly eating triggers (swallowing, eating, smells, and odors, with high cough frequency and even sex split). Cluster 2 comprised those triggered mostly by the need to throat clear and had lower TOPIC questionnaire scores, higher LCQ scores, lower VAS severity, and lower cough frequencies. Cluster 3 comprised those reporting high physical symptom burden who were predominantly female, with low LCQ scores, high VAS severity, and very high TOPIC questionnaire scores. Finally, cluster 4 again was predominantly female and comprised those with high levels of vocal triggers, low LCQ scores, and high cough frequencies. Significant differences were found among these clusters in terms of sex, LCQ scores, TOPIC questionnaire scores, and night VAS severity. Post hoc pairwise comparisons were made to describe differences between individual groups (Table 4).

**Table 4:** Features of Each Cluster Identified in K Means Cluster Analysis

Variable	Cluster 1, Eating Triggers (n = 18)	Cluster 2, Need to Clear Throat (n = 34)	Cluster 3, Severe Physical Consequences (n = 17)	Cluster 4, Vocal Triggers (n = 30)	*P* Value
Sex ratio, female to male	10:8	17:17	13:4	26:4	**.003**^a^
Age, y	57.4 (10.4)	61.3 (14.4)	54.7 (14.9)	60.0 (11.5)	.332
RCC as initial diagnosis	9 (50%)	14 (41%)	8 (47%)	14 (47%)	.933
LCQ					
Total	12.5 (9.7-14.3)	13.4 (11.0-16.5)	6.8 (5.6-9.4)	9.9 (9.3-12.1)	**< .001**^b^
Physical	4.4 (3.8-4.8)	4.8 (4.3-5.4)	2.8 (2.1-3.1)	4.0 (3.3-4.8)	**< .001**^c^
Social	3.9 (3.2-4.6)	4.5 (3.5-4.8)	2.4 (1.3-3.2)	3.0 (2.5-4.3)	**< .001**^d^
Psychological	3.7 (3.3-4.8)	4.6 (3.4-5.5)	2.1 (1.5-2.9)	3.1 (2.6-4.0)	**< .001**^e^
TOPIC questionnaire					
Triggers	14 (14-21)	10 (6-12)	28 (22-32)	17 (14-20)	**< .001**^f^
Sensations	17 (13-19)	12 (8-14)	29 (26-33)	19 (15-22)	**< .001**^g^
Total	33 (28-39)	21 (16-25)	54 (51-64)	35 (31-38)	**< .001**^h^
VAS score, mm					
Day	65 (44-72)	51 (25-68)	75 (48-88)	63 (38-71)	.145
Night	34 (6-60)	32 (2-62)	75 (43-89)	26 (16-39)	**.023**^i^
Verbal cough severity	5 (5-7)	6 (5-8)	7 (5-9)	7 (5-8)	.097
Cough frequency					
24-hour	14.6	10.6	14.2	17.4	.248
Awake	21.8	14.8	19.1	23.2	.309
Asleep	5.0	0.0	0.0	0.0	.424

Data are presented as No. (%), mean (SD), or median (interquartile range) unless otherwise indicated. Cough count data reported as geometric means. Post hoc analysis was conducted using the Mann-Whitney *U* test. Boldface indicates statistically significant values. LCQ = Leicester Cough Questionnaire; RCC = refractory (or unexplained chronic cough); TOPIC = Triggers and Sensations Provoking Cough Questionnaire; VAS = visual analog scale.

a Clusters 1 and 2 were significantly different from clusters 3 and 4 (*P* = .003).

b Clusters 3 and 4 were significantly different (*P* < .001) and clusters 2 and 4 were significantly different (*P* < .001).

c Clusters 3 and 4 were significantly different (*P* < .001) and clusters 2 and 4 were significantly different (*P* < .001).

d Clusters 3 and 4 were significantly different (*P* = .005) and clusters 2 and 4 were significantly different (*P* < .001).

e Clusters 3 and 4 were significantly different (*P* < .001) and clusters 2 and 4 were significantly different (*P* < .001).

f Clusters 1 and 2 were significantly different (*P* < .001), clusters 3 and 4 were significantly different (*P* < .001), and clusters 2 and 4 were significantly different (*P* < .001).

g Clusters 1 and 2 were significantly different (*P* < .001), clusters 3 and 4 were significantly different (*P* < .001), and clusters 2 and 4 were significantly different (*P* < .001).

h Clusters 1 and 2 were significantly different (*P* < .001), clusters 3 and 4 were significantly different (*P* < .001), and clusters 2 and 4 were significantly different (*P* < .001).

i Clusters 3 and 4 were significantly different (*P* < .001).

### Exploratory Factor Analysis and Principal Component Analysis

The Kaiser-Meye-Olkin test gave an acceptable value of 0.779, and Bartlett’s test of sphericity was significant (*P* < .001), demonstrating that the TOPIC questionnaire data were suitable for factor analysis. After removing questionnaire items with a factor loading of < 0.4, a factor analysis was performed. The factor loadings for each component are shown in Table 3; items with a factor loading of < 0.4 were excluded. This confirmed 4 main components that followed the same pattern of sensations and triggers as the cluster analysis (e-Table 2).

## Discussion

To our knowledge, this is the first study to apply the TOPIC questionnaire to evaluate reported sensations and triggers of cough and to assess the relationship with other objective and subjective measures of cough in a cohort of real-world patients attending cough clinics. This study found that TOPIC questionnaire scores were higher in female patients compared with male patients and were not correlated with age. Interestingly, the highest total TOPIC questionnaire scores were associated with greater cough impact and worse QOL as captured by the LCQ, but not with other measures of cough frequency or severity. Hierarchical cluster analysis suggested 4 main clusters of patients based on reported sensations and triggers of coughing captured by the TOPIC questionnaire.

The TOPIC questionnaire was developed based on focus group interviews of not only patients with RCC but also patients with several different diseases in which cough is a pertinent feature. It encompasses a broad range of sensations and triggers that this disease group most commonly reports. Sensations and triggers of cough could suggest differing peripheral or central neuronal mechanisms, or both. By describing the patterns of reported sensations and triggers of cough, phenotypes that may reflect more specific mechanisms can be explored.

To our knowledge, this is the first work to formally describe statistically significant higher frequencies of reported sensations and triggers of cough in female patients compared with male patients as captured by the TOPIC questionnaire. This was coupled with female patients having significantly lower LCQ scores.^12^ This is consistent with previous work in postinfectious cough demonstrating a worse QOL in female patients and work from our group reporting significant sex differences in 24-hour and nocturnal cough frequency.^13^^,^^14^ Reasons for these differences in RCC currently are unknown. However, similar sex differences have been seen in chronic and experimentally evoked pain, with female patients demonstrating decreased conditioned pain modulation compared with male patients.^15^, ^16^, ^17^

TOPIC questionnaire scores within this study did not correlate strongly with commonly used subjective or objective cough measures other than a moderately negative correlation with LCQ scores. This in part may be attributable to overlap in content of the 2 questionnaires, for example, items about embarrassment, lack of control of cough, and coughing during talking.^18^ It also suggests that sensations and triggers of cough impact QOL the most. The weak correlation of TOPIC questionnaire scores with other patient-reported outcome measures and objective cough counts supports the notion that these measures of perceived cough severity and objective cough frequency currently do not capture adequately all aspects of the construct of a patient’s cough symptoms and that new patient-reported outcome measures are needed to profile all elements of a patient’s cough adequately (Table 3).

Data from this study suggest that patients experience different patterns of sensations and triggers and that these fall into 4 main categories or dimensions: severe physical consequences, vocal triggers, eating triggers, and those with a need to clear their throat. Distinct clinical differences were found among these clusters. For example, those reporting severe physical consequences predominantly were female patients with worse severity and QOL. As demonstrated in e-Figure 2, the slope between the third and fourth eigenvalues is less steep. However, the factor loading of component 4 is high (0.866), and the dendrogram also identifies it as a separate cluster (e-Fig 1). Clinically, patients often seek treatment for the need to clear their throat as a cough trigger. On cluster comparison, this group represented a substantial number of the study group (n = 34) and were significantly different to the eating triggers cluster with significantly lower TOPIC questionnaire scores on post hoc analysis. Those reporting the need to throat clear as a predominant symptom experienced poor QOL despite lower cough frequencies. Whether this is the result of differences in peripheral pathologic features between groups or of differing central processing of afferent inputs between individuals with CC remains unclear.^19^

An unexpected finding was the even split of those with a diagnosis of RCC within each cluster (Table 4). Because this study presents baseline data from an initial specialist cough clinic visit, a significant proportion of the patients remain under investigation; therefore, final diagnoses will not be clear until study completion.

Previous studies that have categorized or clustered patients with CC based of sensations and triggers of cough have used dichotomous variables (yes or no answers) about sensations, triggers, or both. The concept of a questionnaire evaluating patient-reported triggers being used to characterize patients with CC was described first by McGarvey et al,^20^ who proposed that two-thirds of predominantly female patients with CC report chemical, thermal, and mechanical cough triggers. Hilton et al^5^ then built on this work to demonstrate that patients with CC can be clustered into 2 groups: patients with a higher number of triggers and lower LCQ scores and patients who report lower levels of cough triggers and higher LCQ score. Neither group showed significant difference in age, cough duration, or reported severity, but the group with lower LCQ scores and higher levels of reported cough triggers were more likely to be female. A further cluster analysis clustered patients into 4 groups predominantly based on sex, age, and cough assessment test score, rather than on reported triggers and sensations.^21^ Koskela et al^22^ also performed cluster analysis on patients reporting cough in a survey of public service employees. The survey was repeated 1 year later to determine cough prognosis. This also demonstrated 2 similar clusters: those with a higher number of reported cough triggers, background disorders associated with cough, lower LCQ score, and longer cough duration and those with lower numbers of reported triggers, cough background disorders, higher LCQ score, and a shorted cough duration. The limitations of this work are the low levels of respondents, the female predisposition of the sample (78% female), and the fact that it was not specific to a population with CC. Also, the cough had resolved for many patients by the second survey.^22^ This work extends previous findings by suggesting more discrete clinical subgroups based on patterns of these triggers and sensations. A further small qualitative study demonstrated that patients do commonly report sensations and triggers of coughing that are categorized into allotussia, hypertussia, and laryngeal paraesthesia; however, this study lacked any control group and did not relate this back to any validated subjective or objective measures of cough.^23^

Previous questionnaires have been developed to assess abnormal sensations in relationship to CC. The Newcastle Laryngeal Hypersensitivity Questionnaire evaluates and categorizes abnormal laryngeal sensations in a range of conditions associated with laryngeal dysfunction, including RCC.^24^ Scores in RCC were different only from those of healthy individuals and were not compared between patients with RCC and those with another cause of cough. The other questionnaire evaluating patient-reported triggers and sensations, the Hull Cough Hypersensitivity Questionnaire, was developed from symptoms reported by a population of patients with significant gastroesophageal reflux disease and reused from a reflux symptom index. Therefore, it does not capture the array of triggers and sensations described in RCC.^25^^,^^26^ It has been evaluated only on its ability to discriminate between RCC and health, rather than RCC and other causes of cough.

Another using the Cough Hypersensitivity Questionnaire included unclear diagnostic groups in its development and validation,^27^ and several participants were people who smoked. The questionnaire also uses dichotomous variables. Through demonstrated patterns of response using a Likert scale in the TOPIC questionnaire, one can pick out dimensions of responses more easily, highlighting the usefulness of this approach. Furthermore, this was a multicenter study recruiting consecutive newly referred patients to 3 specialist cough clinics. The demographic data including sex and age of participants in this study are comparable with those of previous specialist cough clinic populations,^28^ and therefore reflect this population.

This study has several limitations. First, this study reports data only from the initial clinic assessment. Further work needs to be carried out to understand the TOPIC questionnaire response to change after treatment and whether this follows a similar pattern of response within individuals to other subjective and objective measures of cough. The diagnoses used to categorize participants were based on clinician’s first impression; therefore, the prevalence of RCC is likely to be underestimated. Because of the relatively small cohort size, the authors cannot evaluate repeatability of the identified clusters within each facility. This is a cross-sectional study, and hence we cannot directly link TOPIC questionnaire scores to the mechanisms of RCC. Future longitudinal studies are needed to explore how peripheral or central neural blockers influence specific domains of the TOPIC questionnaire score (eg, sensations vs triggers). This could provide critical mechanistic insights, but also prognostic and therapeutic usefulness, but is beyond the scope of this cross-sectional data set.

## Interpretation

A shared feature of many patients with CC is abnormal throat sensations that typically are described as an itch, irritation, or tickle associated with an urge to cough in response to multiple mechanical, thermal, and chemical triggers. This study suggested that these features can be collected in a uniform fashion using the TOPIC questionnaire, which exhibits convergent validity with other measures of cough, especially cough-specific QOL. Moreover, CC phenotypes can be identified by the patterns of sensations and triggers reported. These could point to different neuronal mechanisms and potentially even different treatment outcomes. Further work will explore the ability of the TOPIC questionnaire to respond to interventions, to predict treatment outcomes, and to evaluate if changes in TOPIC questionnaire score correlate with other subjective and objective measures of cough.

## Funding/Support

This study was funded by Merck Sharp & Dohme Corp (grant number MISP 59650). J. K. received administrative support and statistical analysis from the National Institute for Health and Care Research Manchester Biomedical Research Centre. P. A. M. is supported by North West Lung Centre and the National Institute for Health and Care Research Manchester Biomedical Research Centre.

## Financial/Nonfinancial Disclosures

The authors have reported to *CHEST* the following: S. M. P. reports consulting roles with Trevi Therapeutics, Inc., and Merck Sharp & Dohme UK Ltd. K. P. reports travel reimbursement from Sanofi and AstraZeneca UK Limited and speaking and lecture fees from AstraZeneca UK Limited. J. A. S. reports consulting or advisory roles with Wellcome Trust, Merck Sharp & Dohme UK, Ltd., Bellus Health, Inc., Bayer Corporation, Shionogi and Co. Ltd., AstraZeneca UK Limited, Boehringer Ingelheim Corp US, GSK, and Chiesi Pharmaceuticals, Inc.; and speaking and lecture fees from Merck Sharp & Dohme UK Ltd. P. A. M. reports financial support from Merck Sharp & Dohme UK Ltd., speaking and lecture fees from Olympus Corporation, and consulting or advisory roles with GSK. None declared (J. K., S. G., J. W. D., J. M., K. J. H., R. J. D., C. S., J. Y.).

## Supplementary Data

e-Online Data
