# Supplementary material for: Clustering Patients With Chronic Cough Using Reported Sensations and Triggers: Results from the Triggers and Sensations Provoking Coughing Questionnaire
Source: Chest. 2025 Jul 18;168(6):1415–24. doi: 10.1016/j.chest.2025.05.049 (PMC12831080; doi:10.1016/j.chest.2025.05.049)
Supplement: e-Online Data [file mmc1.docx]

**e-Figure 1**


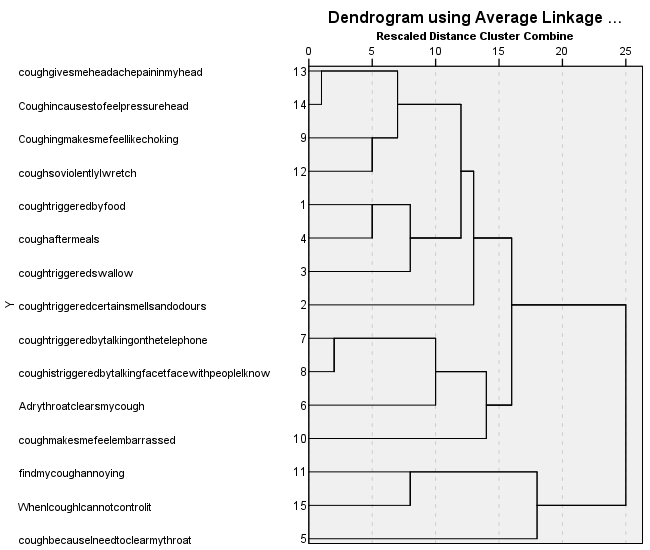


**e-Figure 2**


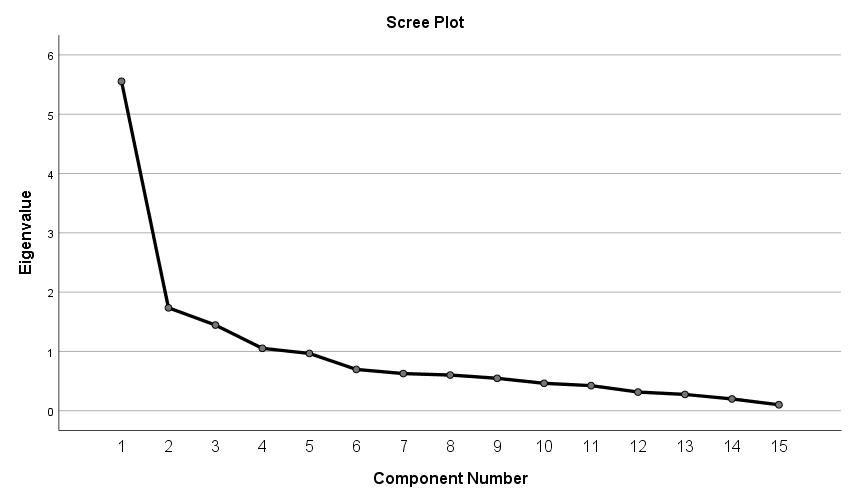


**e-Table 1**

**
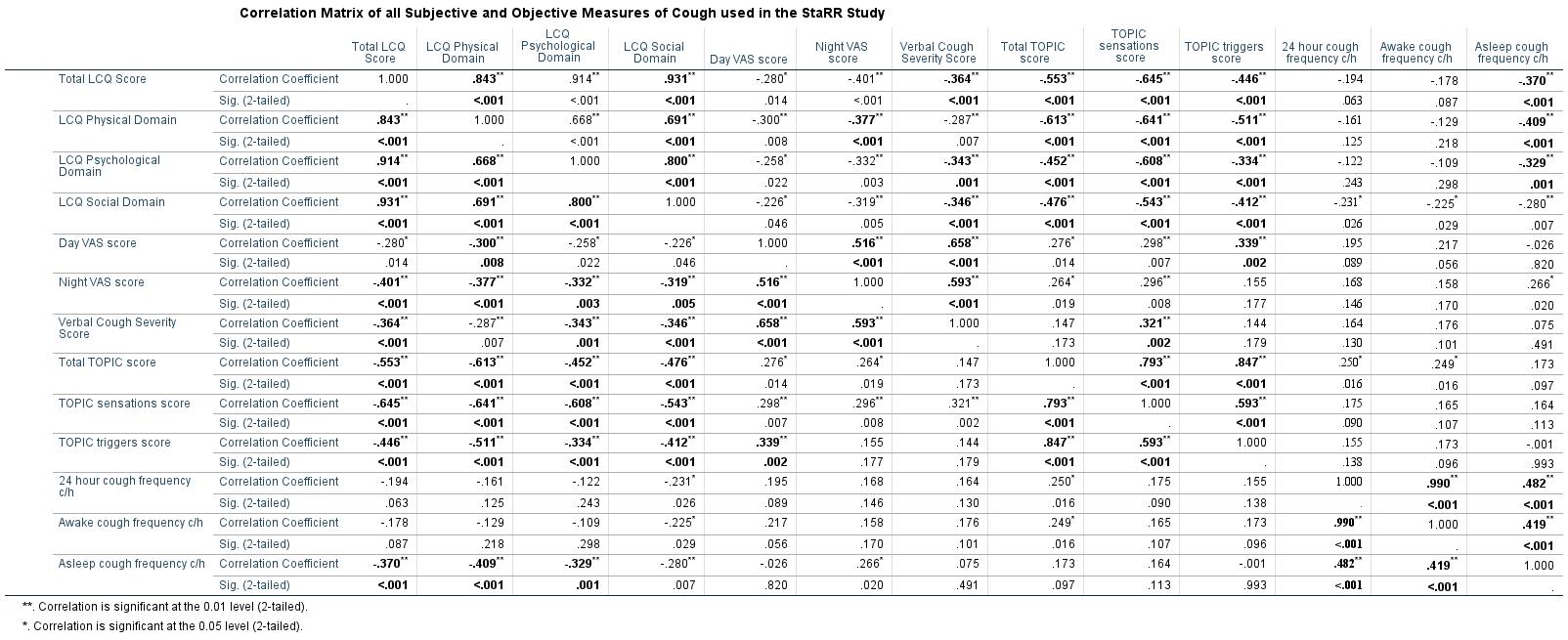
**

Significant correlations in bold.

Correlations performed using Spearman's rank correlation coefficient.

**e-Table 2**

| **TOPIC Questionnaire Item** | **Component** | | | |
| --- | --- | --- | --- | --- |
|  | **1**  Severe Physical consequences | **2**  Vocal Triggers | **3**  Eating Triggers | **4**  Need to clear throat |
| **Pressure in head** | 0.876 |  |  |  |
| **Headache** | 0.858 |  |  |  |
| **Retching** | 0.774 |  |  |  |
| **Choking** | 0.562 |  |  |  |
| **Cannot control** | 0.466 |  | 0.471 |  |
| **Talking F2f** |  | 0.852 |  |  |
| **Talking on phone** |  | 0.841 |  |  |
| **Dry throat** |  | 0.666 |  |  |
| **Annoying** |  | 0.485 |  |  |
| **Embarrassed** |  | 0.61 |  |  |
| **Foods** |  |  | 0.861 |  |
| **After meals** |  |  | 0.854 |  |
| **Swallow** |  |  | 0.536 |  |
| **Smells and odours** |  |  | 0.504 |  |
| **Need to Throat clear** |  |  |  | 0.866 |
